# Agent models of customer journeys on retail high streets

**DOI:** 10.1007/s11403-022-00350-z

**Published:** 2022-05-09

**Authors:** Paul M. Torrens

**Affiliations:** Department of Computer Science and Engineering, New York University, Floor 13, 370 Jay St, Brooklyn, NY 11201 USA

**Keywords:** Retail, Agent-based model, Customer journey, C53, C63, C8, D03, D12, L81

## Abstract

In this review paper, we aim to make the case that a concept from retail analytics and marketing—the *customer journey*—can provide promising new frameworks and support for agent-based modeling, with a broad range of potential applications to high-resolution and high-fidelity simulation of dynamic phenomena on urban high streets. Although not the central focus of the review, we consider agent-based modeling of retail high streets against a backdrop of broader debate about downtown vitality and revitalization, amid a climate of economic challenges for brick-and-mortar retail. In particular, we consider how agent-based modeling, supported by insights from consideration of indoor shopping, can provide planning and decision support in outdoor high street settings. Our review considers abstractions of customers through conceptual modeling and customer typology, as well as abstractions of retailing as stationary and mobile. We examine high-level agency of shop choice and selection, as well as low-level agency centered on perception and cognition. Customer journeys are most often trips through geography; we therefore review path-planning, generation of foot traffic, wayfinding, steering, and locomotion. On busy high streets, journeys also manifest within crowd motifs; we thus review proximity, group dynamics, and sociality. Many customer journeys along retail high streets are dynamic, and customers will shift their journeys as they come into contact with experiences and service offerings. To address this, we specifically consider treatment of time and timing in agent-based models. We also examine sites for customer journeys, looking in particular at how agent-based models can provide support for the analysis of atmospherics, artifacts, and location-based services. Finally, we examine staff-side agency, considering store staff as potential agents outdoors; and we look at work to build agent-based models of fraud from customer journey analysis.

## Introduction

For a long time, the vibrance of retail on high streets has persisted as a focus in discussions about urban vitality (Bunting and Millward 1998; De Nisco and Warnaby 2013; Dolega and Lord 2020; Millward and Bunting 1999; Robertson 1983, 1997; Sternlieb 1963; Warnaby and Medway 2016; Weisbrod and Pollakowski 1984). The connection between high streets and downtown success is often framed by the significant influence that retailing plays in local community building (Cotton and Cachon 2007; De Nisco and Warnaby 2013; Kohijoki 2011; O'Brien and Harris 1991; Talen 1999) and in local economies (Lewis 2001; McCall 2011; Zhang et al. 2020). Retail high streets are often a central fixture in urban planning and policy debates about how we should plan and manage the future of our cities. Retail high streets feature in discussions about compact cities (Gordon and Richardson 1997), the linear city (Batty 2022a), coherent cities (Salingaros 2000), smart cities (Kitchin 2014), and the 15-min city (O'Sullivan and Bliss 2020). High streets are also now a central focus of the emerging debate about the post-pandemic city (Batty 2022b; Moreno et al. 2021). In many cities, retailing along high streets is intricately bound to consideration of development density, housing, and access to transport, as in the case of considerations of transit-oriented design (Cervero 1996; Cervero and Kockelman 1997) and new urbanism (Duany et al. 2001; Katz 1993; Talen 2002). In these situations, the interplay between urban design factors and flow of customer traffic through retail high streets is a significant component in determining general accessibility and foot traffic at the hyper-local scale underneath larger urban designs and plans (Hess et al. 1999; Moudon et al. 1997). In short, how and why people move around, outdoors, on streetscapes provides the dynamics that energize broader discussions of urban planning, policy, and design.

Retail high streets typically present as centrally-situated corridors of store fronts, usually flanking sidewalks along main transport arterials in cities, and they are commonly anchored to (and by) focal urban and public infrastructures, such as transit, office buildings, and municipal services (Couclelis et al. 1987; Pyle 1926). In many cities, retail high streets are a significant element of what forms the *streetscape*, i.e., the visible and accessible spaces that form between buildings, sidewalks, and roads. High streets are uniquely important in many social science considerations of urban form, because they represent one of the most significant interfaces between public and private space in cities. Retail high streets traditionally support a huge flux of people, goods, and transactions in urban downtowns. Alas, recently, there have been growing incidences of relative decline in the fortunes of retail high streets (Peterson 2017). The reasoning behind this ebb is broadly debated, but seems to be sourced in a combination of factors that include changes in customer shopping habits, a general downturn in the appeal of the downtown as a site for activity in some cities, competition from e-commerce (Berman 2019; Byun et al. 2020; Ives et al. 2019; Zimmerman 2012), and a recent, rather marked, and quite sudden collapse of physical retailing during the COVID-19 pandemic and restrictions on public gatherings (Forsythe et al. 2020; Roggeveen and Sethuraman 2020).

Emerging patterns of relative decline in the fortune of retail high streets have increased the potential necessity of modeling and simulation as media for examining what-if scenarios for decision- and planning-support. Computer modeling and simulation are often crucial in consideration about the future of the high street and the downtowns that host them, as tools that allow stakeholders to examine potential pathways for urban futures, and as media for proactively assessing the viability of assumptions, hypotheses, plans, and policies (Batty et al. 2001). Agent-based modeling, as a particular simulation methodology, has emerged as a significant medium for urban simulation, due primarily to the natural affinity between agents and high-resolution representation of cities at scales that range from individuals and their moment-by-moment dynamics, through to the functioning of coarse-scale phenomena such as crowd flow, traffic congestion, and location dynamics (Benenson and Torrens 2004; Heppenstall et al. 2012). Agent-based models are voracious in their appetites for data, particularly for applications at the scale of urban downtowns, which require unprecedented detail and resolution to hope to resolve (Torrens 2012). High-quality and high-resolution data for downtown models have long been hard to come by, with the result that simulations are often built from limiting assumptions that constrain the ability of models to support plans, policies, and decisions with parity to real-world concerns (Batty and Torrens 2001). Examples of these assumptions include substituting sudden displacement of pedestrians for their movement decisions (Blue and Adler 2001), the use of physics to replace behavior (Helbing and Molnár 1995), and assumptions of boundedly rational action in human decisions about where to go and what to do in downtown environments (Arthur 1994; Simon 1956; Zhu and Timmermans 2011). These limitations and short-cuts are quite simply unavoidable in many cases (Epstein 2008), as there are usually not sufficient data available to do otherwise, because the underlying perception and cognition that drives behavior is not observable and is often unreliably revealed by case studies or survey instruments (Louviere et al. 2000).

However, data constraints on agent-based simulation are loosening, due in large part to the emergence of sensing technology and related information systems for building knowledge through automated means (Torrens 2016a). Indeed, retailing and data analytics for customer experience—sometimes referred to as “retaillance” (a portmanteau of retail and surveillance)—have been at the forefront of these developments (Elnahla and Neilson 2021). In this paper, we will review how retail intelligence technologies, in particular, are revealing very detailed and broad insights into how people act, interact, and transact in urban economic spaces. We argue that much of this technology could be usefully considered for use in agent-based modeling, with the potential to better ground agent simulations to real-world dynamics of streetscapes. In particular, we consider the concept of the customer journey in retail analytics and assess it as a framework for studying high street dynamics. Our focus, initially, is on applications to high-resolution urban geography, primarily because we have identified geography as a useful supporting framework for customer journeys. However, we also see broad potential for uptake in urban economics more generally.

The key take-aways for the reader will be as follows:

- The retail framework for considering a customer journey through indoor retail environments and services can be usefully adapted to shed new insight into pedestrians’ movement through outdoor urban geographies and economic geographies of the high street;
- In particular, the delineation of customer experience into journey paths, service-scapes, and touchpoints that connect them can be useful concepts for structuring consideration of how people come into contact with urban environments;
- Geography provides a natural structure for framing customer journeys, and for extending the concept to outdoor settings;
- A variety of methods for data-gathering and data analysis are available from retail science that can empirically inform our understanding of how people perceive their journeys through high streets and how they build cognition and knowledge as they move through cities;
- Agent-based models could straightforwardly make use of these data to support a wide array of simulation scenarios for retailing, both indoors and outdoors, as well as for broader consideration of urban phenomena at the streetscape;
- Retailers’ knowledge of the interchangeability between cyberspaces of e-commerce and tangible spaces of retail stores suggest novel pathways for scholars in the social sciences to consider new data-sets and perspectives on human behavior that takes place in the “omnichannel” between those spaces;
- Automation in retail intelligence for the customer journey, particularly through connections to retail information systems, suggests new options for connecting observations of pedestrian behavior to agent-based models in ways that could efficiently scale to large-area studies of downtown environments;
- Retail analytics raise some serious concerns about privacy and surveillance, particularly as we consider that technologies and methods designed for indoor private spaces could be transferred with little modification outdoors and into the public realm.
- In the near-term, as many cities grapple with how to recover from sudden declines in the fortune of retail high streets due to the COVID-19 pandemic, considerations of how cities and community groups can plan and manage for outdoor customer journeys may draw customer journey research into closer alignment with agent-based modeling and urban policy.

## Customer journeys as paths through urban geography

Retailers across many different enterprise segments have long valued high street locations due to the numerous overlapping geographical advantages that access to the street affords them (Brown 1994). Perhaps chief among these benefits is the ability of a store to place its retail operations in incredibly close proximity to a steady and reliable stream of would-be customers that have been drawn to the high street to either shop or to engage in other economic activities. Rents for high street locations are of course expensive compared to other locations for this reason. For example, Zhang et al. (2020) recently showed that the average rent in a shopping district in the Netherlands is ~ 70% higher per square meter than it would otherwise be in a non-shopping district and that this gap remains relatively constant even as the macro-market for rents shift (p. 32). Nevertheless, despite the upfront costs, a high street storefront affords retailers an ongoing opportunity to essentially skip much of the guesswork that would normally be necessary to engage shoppers from the background of the general population (Harris et al. 2005), and to thereby sidestep acquisition costs required to draw shoppers to the vicinity of their stores.

On a high street, then, retailers can focus on competing for (and catering to) the attention of people that are already of reasonable likelihood to be shoppers. Many stores are in competition for this foot traffic on any given retail high street, often over close quarters. Brown (1994) stated the problem with eloquence when he mentioned that, “a few yards can make all the difference between success and failure in retailing” (p. 543). High street retailers may focus considerable effort in swaying passing pedestrians to shop in their stores. To do so effectively, retailers must often discern who (from among the passing pedestrian footfall) they would like to entice to patronize their stores, then they must figure out how to steer those people through their doors. Inevitably, these twin problems of identifying would-be customers and drawing them into the store are intertwined with the *customer journey* that unfolds along the high street and the myriad factors (retail-specific and otherwise) that shape those journeys for individual pedestrians. In this paper, we argue that these are also the types of problem statements that agent-based modeling is apt to address in simulation. Specifically, the dual challenge of spotlighting individual agency within a motif of collective agency and against a dynamic backdrop of multi-scale complexity is one that both retail service provision and agent-based modeling are well equipped to address.

Customer journeys are usually considered indoors. Within retail premises, retailers have a great deal of understanding about what services they can marshal to meet customer needs and they may often have local knowledge of customer habits and trends. In-store, retailers may also collect significant empirical data, for situations such as product placement for which they have a lot of experimental control. Nevertheless, customers will never even come into contact with in-store resources until they walk through the store doors. A *huge* portion of the customer journey actually takes place outside stores, and much of it unfolds on the retail high street. Again, this is why retailers site their facilities on high streets, at fantastic cost to their operations. The customer journey along high streets is incredibly relevant to retailers, but it is difficult to study. Retailers’ ability to collect data about customers, and their options for experimentation diminish rapidly outside their walls. While high street retailers can be generally assured that their customers are passing by on the sidewalks outside, retailers lack the sorts of fine-scale detail that would allow them to target those customers with service offerings. This stands in almost direct contrast with Online retailing, in which every click, hover, and swipe that a customer makes along their virtual journey is potentially known. Material retail stores are increasingly in tremendous competition with Online retailers, partially because of the relative informational imbalance between them (Grover and Teng 2001). Indeed, many e-commerce platforms have widespread capabilities to build hyper-detailed profiles of visitors, customers, and the journeys that take them to and through their platforms (Wiedmann et al. 2002).

In this paper, we will make the case for agent-based modeling as a tool for generating actionable information about the customer journey on retail high streets, in ways that cast the customer journey as a framework beyond store walls. The aims of this paper are to reveal key aspects of the outdoor customer journey on retail high streets, and to map those traits to agent-based modeling methods. In service of these aims, we review the existing literature for indoor customer journey determination and analysis, the burgeoning research being done to build understanding of outdoor customer journeys, agent-based modeling for retail support, and aspects of modeling for simulating outdoor pedestrian movement, which has several points of potential crossover utility for high street modeling in general. In a bid to provide a roadmap to support tighter connections between factors of the customer journey and agent-based modeling, we conclude by discussing how data science could better support outdoor customer journey analysis.

## Customer journeys within retail operations

Let us first consider how retailers frame the customer journey within stores, before examining how the concept might be broadened to the outdoor environment of the retail high street. The customer journey concept extends the related idea of ***customer experience*** (Voorhees et al. 2017). Studies of customer experience are (generally) concerned with how retailers may manage the processes of service delivery, (alongside the products being sold) to co-create an environment for consumption with the customer. We refer to this consumption environment hereafter as a ***service-scape*** (Bitner 1992; Clarke and Schmidt 1995; Hall 2008; Johnstone 2012). Within a customer experience, a ***customer journey*** is considered as the cumulative set of steps that a customer takes from pre-purchase, through purchase, and on to post-purchase interactions with a retailer. The customer journey concept specifically considers the points of contact that are invoked between customers and the retail service offerings (rather than, say, a would-be customer’s motivation to shop). A ***customer journey path*** considers a specific sequence of contacts that customers encounter in their journey through service offerings. These points of contact are considered as ***touchpoints*** (Ieva and Ziliani 2018), e.g., passing by a storefront and examining the display, accepting an invitation from a staff member to try a sample, repeatedly passing by street signs that advertise a store sale, etc. Touchpoints may be physical, they may be virtual (e.g., receiving a digital coupon on your smart device when you near a store), or they may manifest as combinations of both (e.g., scanning a Quick Response (QR) code on a flyer that is handed to you at a high street train station and directs you to a store location nearby). This combination of material and virtual touchpoints relates to the retail “***omnichannel***” (Barwitz and Maas 2018), the idea that retailers might offer services that allow a customer journey to start in an e-commerce channel, but switch to material commerce (or vice-versa).

The customer journey path is a highly significant component of how retailers consider the processes underpinning their services. The path may influence staffing, the placement of facilities, orchestration of loss prevention, design of lighting, and display configuration, among other considerations. Retailers are often interested in building empirical data about the customer journey path, which they may use to project their service plans. In some instances, retailers may have ***service blueprints*** that they work from when projecting customer experiences to their service offerings (Bitner et al. 2008; Patrício et al. 2011). Service blueprints may take on many forms, but usually represent a set of formal service plans, either for a whole store, a store process (e.g., deployment of sales staff, how to handle curbside delivery, how to service outside seating), or perhaps particular customer types (e.g., customers with elevated customer loyalty status, first-time visitors to a store, convenience customers that require fast checkout, etc.). A ***customer journey map*** allows a retailer to formalize (and to visualize) how a typical customer might encounter a store’s services; the map serves to essentially juxtapose canonical customer behavior relative to the service blueprint that a retailer has to support them. In some cases, customer journey maps are actual maps (Larson et al. 2005; Rosenbaum et al. 2017): store layouts with product offerings and the locations of checkouts, and some notation of the typical trail that a customer might take through them, if convenience shopping as opposed to shopping for weekly or monthly household needs, for example. The former might chart a typical grab-and-go type journey, which would prompt the placement of convenience consumer packaged goods near checkouts. The latter could suggest a store layout that sites household staples such as bread and milk in extreme corners of the layout, with counter-serviced goods such as bakery items strategically placed in between to route journeys past end-of-aisle displays (Tan et al. 2018).

At its core, then, the concept of the customer journey centers on considering the journey that a retailer can discern from ***bundles of customer paths***, relative to service offerings. The juxtaposition of journeys relative to retail service-scapes form what Thomas et al. (2020) referred to as “fields of alignment” (p. 9). How well the journey and service-scape align is a useful consideration, particularly if we also consider that many customer journeys should (or do) come into windows of alignment at touchpoints. Figuring out this alignment is not always straightforward. The bundles that customer journey paths form will vary depending on the retail services being considered (e.g., compare customer journeys for restaurants compared to those for fast food), and may constitute idealized customer experiences, average customer interactions with services, or the paths generally taken by specific profiles of customers. It is also feasible that one could consider singular customer journeys (and this is something that agent-based modeling may be able to facilitate, at least for synthetic customers). While it may be efficient for retailers to build their service-scapes around a few canonical customer journey types, the reality is that there is likely a very wide diversity in customer path types (as there are customer types). And, the fields of alignment that form between paths and service-scapes may more often manifest as a dynamic economic geography that is co-determined between the customer and the various service levers that a store may use to create an advantage, say, from physical space and brand space, for example. Bartelheimer et al. (2018) mentioned that “retailers cannot view single service encounters in isolation but have to consider their impact on the overall customer experience creation” (p. 3). Multi-agent modeling, in which the interactions between different types of agents and agency in an environmental or systems context, would therefore seem to be a very useful tool for evaluating the co-creation of customer experience. Indeed, there is a growing consensus that customer journeys and service-scapes are increasingly co-produced by retailers and customers, and that the omnichannel is wresting some of those production factors away from retailers because of social media for ratings, review, and product advocacy (Kotler 2010).

## Framing customer journeys as paths through retail geography

In this paper, we would like to suggest that the ***geographical paths*** that customer take while engaging in the customer journey might form a useful organizing principle for considering retail operations, as well as for considering how insight from retail analytics of customer journeys might influence agent-based modeling and applications to high streets. (We will also go on to make the follow-on argument for using agent-based modeling to generate those paths in simulation, as a tool for retail planning and decision support.) Our reasoning is as follows.

If we isolate the customer experience to a geographical path, then various geographical viewpoints can be brought to bear on contextualizing that path (O'Brien and Harris 1991) and this is useful for high street retailing, in particular, for which retailers must often try to pinpoint customer and would-be customer journeys from the moment-to-moment rhythms and motifs of everyday streetscape scenes. Specifically, we may invoke *behavioral geography* to examine customer journeys from the perspective of individual shoppers and their perception and cognition of the retail high street service-scape (Brown 1988; Coshall 1985; Golledge and Timmermans 1990; Kurose et al. 2001). We may use *urban geography* to examine how locations of stores along a given high street segment might explain foot traffic dynamics and the characteristic paths of individual customers within that traffic stream (Borgers and Timmermans 1986a, 1986b; Hahm et al. 2017; Van Der Hagen et al. 1991; Weisbrod and Pollakowski 1984). One might consider the *economic geography* of a retail high street to assess the spaces of complementarity and competition for customer journeys among retailers or between retailing and other economic activity on a high street (Dawson 1988; Laulajainen and Gadde 1986; Scott 1970). One could contemplate how *human geography* can frame the social and cultural spaces and places that manifest on high streets and within pedestrian crowds and what roles retail services play in those phenomena, for example in influencing fashion (Crewe 2010), peer influence (Stevens et al. 2019), and the relationship between value platforms and consumption (Crang 1996; Crewe 2000; Evans 2020; Goss 2004). Regarding the omnichannel, in particular, much of the work in *cybergeography* (Adams 1997; Batty 1997; Dodge 2001) has spotlighted new ideas about how material and virtual retail geographies are co-determined (Dodge and Kitchin 2005; Graham 2005; Kirsch 1995; Kitchin and Dodge 2011; Thrift and French 2002).

We see some immediately practical implications for viewing customer journeys as paths through retail geographies. For example, in considering how service blueprints and customer journeys align, one might isolate the ***physical path*** of locomotion and movement that customers take through stores’ service offerings (Lee et al. 2013), including the configuration of that path through stages of the shopping task, from store entry to promotional spaces, past shelves, to queuing areas, through checkout, and so on. Physical paths play out via consumer distance and through floor space (which are critical elements of stores’ fixed costs), but also in the time that customers must devote to traversing that space. One might also then recognize the ***time geography*** associated to a physical path (Nara and Torrens 2007, 2011; Timmermans et al. 2002; Van Der Hagen et al. 1991). Consider for example the contrast in time geography of retail service spaces that are designed to encourage customers to lose track of time in-store (as is the case in casino type environments as well as in popular warehouse-like self-assembly furnished goods stores), versus spaces that are designed to facilitate very rapid-service dynamics in each and every square meter of floor space (as in fast food restaurants, for example). In some cases, the mapping between physical paths and time geography is used quite blatantly to the retailers’ advantage, as when grocers place high-temptation goods such as candy at the point of checkout, where the customer path physically narrows to a queue and time geography crawls to a sink. One may also consider the ***social path*** that customers traverse as they move past and with other shoppers and store staff (Thomas et al. 2020), particularly the peer effects, biases, and influences that social interactions can effect and how these may be used as part of a retail service offering (Crewe 2000, 2001, 2003). Other examples can be seen in how food retailers make use of human geography when encouraging groups of young customers to sit in outdoor seating in the early evening to yield a social impression for a restaurant, or how they might organize high-profit-margin products around tables in a consumer computing goods store to yield a crowd effect of peer buyers’ tangibly playing with a newly-released product. Retailers of course spend considerable time honing the ***mediated path*** that customers take past displays, product placements, and advertising media (Adams 2011; Bhargava and Donthu 1999). And one must also consider that alongside the aforementioned paths, customers also traverse ***paths of choice and decision-making*** to arrive at consumption decisions (Dogu and Erkip 2000; Gibson 1966; Golledge 1978; Penn 2003; Stern and Portugali 1999), e.g., how spatial cognition (Golledge and Stimson 1997) might relate to the retail sales funnel that customers may descend through, beginning with awareness, passing through interaction with the retailer, and ending in a purchase and post-purchase engagement such as brand loyalty or review (Fulgoni 2014). One of the main benefits of a geographical perspective on high street retailing is that it often ***scales***. Within a general pattern of movement and interaction along a high street, for example, customer journey paths could be isolated to fine-scale and high-resolution factors surrounding characteristics of customer types and shopping types. (This is bidirectional in some cases: for example, we might consider the paths that customer categories take for given shopping actions.) Millonig and Gartner (2011) have described a massive project to do exactly this, typifying both customer types and shopping actions from traces of movement data that customers cast while engaged in their journeys along high streets. Similarly, coarse-scale and lower-resolution conceptualizations of the customer journey could be considered, for example by discerning shopping trips from within the broader dynamics of diurnal activity in a downtown (Anderson 1971; Brail and Chapin Jr 1973; Chen et al. 2011; Goodchild and Janelle 1984; Janelle et al. 1998; Robertson 1983).

At the touchpoint, the path of the customer journey comes into direct relevance with the service-scape and the retail operations that are associated, e.g., staffing, product display, lighting, payment technology, etc. Among many authors, there is a growing appreciation that the touchpoint is actually a point of value *co*-creation (between the retailer and the customer). As Bartelheimer et al (2018) explains, “During these episodes of contact, both parties integrate their resources such as the customer’s preferences and the retailer’s knowledge to provide service to each other.” (p. 3). Moreover, value co-creation can also involve *context* drawn from the site of the touchpoint. For example, Berendes et al. (2018) discussed the significance of examining the precursors (p. 220) to touchpoint events: the aspects of the customer journey that may lead a would-be customer to the touchpoint. In this sense, context could include social factors of other customers through peer influence, technological cross-over through the omnichannel (e.g., store reviews available Online), and value propositions that are invoked from marketing (Verhoef et al. 2009). This context may take place at a given touchpoint, but its origins may come from factors that can be tangential to the situation of that touchpoint (Schmidt et al. 1999). At the finest resolution of geography, then, customer journeys interact and possibly transact with retail touchpoints through co-incidence of space and time, and the geographical context in which the space and time comes into consideration. This co-incidence—and the geographical context—may be material, or it might be virtual. Considerations of the geography involved can accommodate both perspectives. Berendes et al. (2018) raised the issue that, “Customer have myriads of digital and physical touchpoints with retailers” (p. 218) and that they may alternate between these modes (channels) along the customer journey. This, they point out, “transforms customer journeys into sequences of intermingling online and offline service encounters” (p. 219). It also presents an opportunity for collecting data along the customer journey as the omnichannel for retailing provides access to digital touchpoints that may have co-expression in tangible aspects of the service-scape. However, it would seem perhaps straightforward to argue that digital data regarding physical touchpoints can only ever serve as a proxy for the actual lived experience in the real world.

## The challenges of examining customer experience outdoors

The concept of the customer journey is reasonably well understood in-store, where retailers have considerable latitude to experiment with services and to learn about what works well (or not). This experimental leeway supports a wide range of theoretical propositions from diverse academic fields. However, the notion of the customer journey is not as frequently considered beyond the store, despite the fact that retailers rely heavily on traffic that comes from the high street. This is because of difficulties in gathering information and in making sense of what is going on at the macro-scape of the high street.

The first challenge in considering outdoor customer journeys is that it is difficult to figure out why customers behave the way that they do, and incredibly harder to do so when you have little information about what they are doing. Retailers know an exquisite amount of facts about customers that walk through their doors; but less about those that do not.

A second problem with understanding high streets stems from retailers’ lack of information regarding the features of high streets and customers’ interactions with them. Stores usually know a lot about the shop floor environment that hosts their products and over which their staff operate. Similarly, most people that walk through a store’s doors may be considered as customers. Retailers cannot make the same assumptions outside. Retailers do not know if pedestrians on a sidewalk outside their stores are actually customers; and they often have imperfect knowledge about the features of the high street, which can change all the time as vans pull up to curbs and start unloading goods, as queues form at bus stops, as rain puddles form on sections of the sidewalk, and so on.

A third difficulty is that unlike in-store operations, much of the action on the high street is beyond the direct and operational control of retailers. Retailers may be able to cast lighting onto the sidewalk and install signs and advertising on their store fronts, but there is little else that they can do to modify the high street around their stores. Retailers have considerably less degrees of freedom in shaping the customer experience outdoors—on a retail high street—than they would have within their own stores. Afterall, streets are public spaces and people have latitude to come and go as they please, and sidewalks are first and foremost designed to support walkers trying to get from one place to another. This makes it challenging for retailers to *extend* their existing store operations outwards, onto the high street. Any physical assets that retailers place on the street are exposed to theft and the elements, and retailers must usually be careful not to interrupt pedestrian flow, e.g., when placing storefront displays, sales racks and counters, outdoor seating, etc.

A fourth issue stems from the fact that much of the tangible dynamics of custom, competition, and complementarity that takes place on high streets is even opaque to most retailers’ *view*. In some senses, it is difficult for retailers to know what to look for on the high street. The general idea that high street locations are good for business is holistically understood and well covered in the literature already (Dawson 1988; UK Ministry of Housing Communities & Local Government 2018). However, specificity in exactly how those locational benefits accrue to individual retail operations has long been difficult to ascertain at micro-scale [this is very well discussed by Brown (1987, 1994)].

Around these four principal challenges to examining customer journeys outdoors, it is worthwhile to also recognize that, in many ways, tangible retailing on the high street has steadily been losing ground to e-commerce. As customers have increasingly begun to use their smart devices while outdoors, many of the location-aware technologies (Hazas et al. 2004) and the location-based services (Junglas and Watson 2008) that have sprung up around their information search behavior (Watson et al. 2002), their tagging of things that they see and interact with, their consultation with mapping and navigation services (White et al. 2000), and their movement and locomotion (e.g., fitness apps) have fused with e-commerce (Dhar and Varshney 2011; Fano 1998). Considered in the theme of this paper, much of the touchpoint and journey activity that customers partake in while on retail high streets has already been coopted by e-commerce platforms. Indeed, in some cases, large Online retailers have begun to build test stores on high streets that essentially substitute much of the store service-scape to technology: *Amazon Go* (Ives et al. 2019; Wingfield 2018) is a well-known example in the USA; *Alibaba*’s *Hema* stores (Fannin 2018) are an equivalent in Asia. Traditional physical stores on high streets have been caught flat-footed by these developments, largely due to the inherent myopia that they face when seeking insight from the high streets around them. In contrast to the increasingly exquisite sapience and configurational control that retailers have within their stores, retail capabilities to shape the customer experience taper-off abruptly at the front door of their shops, where the private and commercial space of their operations give way to the public commons of the streetscape outside. This near-sightedness is particularly evident for retailers that are seeking to understand and map their operations to manage the customer journey.

High street retailers are therefore often forced into a reactive relationship with the high street, in which they can merely chase general rhythms and motifs from high street dynamics for signals that they might map to their operations. This can leave huge portions of retailers’ business models essentially “fuzzy,” and open to vulnerabilities for misaligned investment. This is unusual for an industry that is generally considered as laser-focused on profit margins. Edelman and Singer (2015) state this quite well when they write that “companies have been reacting to customers, trying to anticipate their next moves and position themselves in shoppers’ paths…” (p. 90). To some extent this reactive approach represents a sort of myopia that contrasts with the keen, fine-resolution focus that is available in the foreground of their in-store operations. Thus, while in-store (and cross-channel) retail operations represent some of the most data-rich environments in the business world, retailers are often left in the dark as to how effective their decisions are on the high street at their front doors.

## Using agents to model the customer journey on high streets

Retailers have a long-standing interest in modeling and in simulating the customer journey. For retailers, ***modeling*** may involve the use of a concept model, a data model, or a process model to frame the customer journey relative to their store operations and to parameterize relevant components of the customer journey, for example relative to key performance indicators (KPIs). ***Simulation*** is perhaps less widely used by retailers in their day-to-day operations. Simulation facilitates the exploration of what-if questions relative to a model (e.g., questions such as what if new products are introduced, what if a store alters its entrances and exits, what if an advertising campaign is launched to target a new customer demographic?), as well as to fill-in gaps in the model. The latter is very relevant to our discussion here, as a common gap in retailers’ control is the customer journey: a store may realign its layout, but until an actual customer begins to interact with that new design, the retailer cannot reasonably assess what implications result. This is where simulation scenarios based on ***agent-based models*** can come into particular usefulness, in allowing the retailer to experiment with synthetic versions of the customers that they seek to influence (whose custom habits are not guaranteed), and approaches that they might have available within their store operations (e.g., staffing, opening hours, location decisions, etc.) for which they do have tangible control. In particular, examination of customer journeys through agent-based modeling allows potentialities of those journeys to be conveniently subjected to experimental pipelines that move from theory through concept to data and modeling to simulation and what-if scenario building. Each part of this pipeline is useful in contextualizing the customer journey against (costly) retail operations, and in connecting the customer journey maps to service blueprints.

Ali and Moulin (2005), in their paper on modeling shopper behavior in indoor malls, mentioned a concern that permeates much of the agent-based modeling literature and that extends to modeling of customer journeys. That concern is worth repeating here: that “there are few MAS-based research studies attempting to simulate human ‘knowledge-based’ behaviors in micro-scale geographic environments (e.g., malls, shops, hotels, airports, etc.).” (p. 445). Ali and Moulin (2005) go on to argue that personal characteristics and spatial behaviors of individual shoppers work hand-in-glove with features of mall environments (in their example) to produce shopping behavior (p. 446). Batty et al. (2003) raised a similar point, arguing that at fine-scale resolutions of streets, people begin to identify “more closely with elements nearer to our everyday experiences” (p. 674). Batty et al. (2003) also discussed how movement may shift in its character *at* different scales (p. 675).

It is apparent, then, that the customer journey actually comprises quite distinct paths and purposes when considered at different scales. In our review, therefore, we focus on what might be achieved to develop models of the customer journey from the standpoint of *individual agency*.

### (Purely) conceptual models

As we discussed in Sect. 3, the service blueprint is often the primary concept model that retailers utilize when considering how the customer journey may map to the offerings embedded in a service-scape. There may be as many service blueprints in use as there are retailers, but chain stores at least may have a common service blueprint to standardize the customer experience across their many locations. In many cases, a retailer may even tie changes in the service-scape to KPIs via the customer journey. In this way, the customer journey becomes the vehicle that animates the service blueprint and may be considered, purely conceptually, as “agent-adjacent.” (It is also therefore problematic that there are relatively few retail KPIs for the high street outdoors and that large portions of the customer experience along streets in opaque to retailers’ insight.)

Work by Titus and Everett (1995) introduced an incredibly detailed conceptual model of the Consumer Retail Search Process (CRSP), which constitutes portions of the customer journey. A critical contribution of their work was to classify search behavior on the basis of *environmental support structures* and *drivers from consumer behavior*. Environmental factors included environmental differentiation, environmental visibility, orientation aids, and spatial configuration (p. 108). Consumer behavior included the characteristics of individual customers or customer types, based on environmental search knowledge and sensitivity, environmental perception, navigational strategy, movement, contact, time pressure, task complexity, and considerations of search satisfaction based on effectiveness and efficiency (p. 108). Titus and Everett (1995) introduced these concepts for indoor retailing, but tailoring them for the outdoors would not involve much retooling of the concept (although feeding data to those concepts for high street settings would be challenging.) Indeed, we can consider that agent-based models could be useful in animating dynamics (and generating synthetic data) atop the types of conceptual models represented in service blueprints or the CRSP introduced by Titus and Everett (1995). In particular, agent-based simulation scenarios could provide (1) what-if experimentation with service-scape design, as well as (2) synthetic scenarios to examine unknown parts of the customer journey in ways that allow retailers to plan for the unseen and unforeseen components of high street dynamics. Aspects of this work have been demonstrated by others through agent-based approaches, albeit over a scattered literature and for applications that are initially removed from retailing. In what follows, we review how one might go about mapping key components and concepts of the customer journey to agents.

### Non-stationary retailing

A critical consideration in examining customer journeys through retail high streets is that many vendors and shopping opportunities are not stationary on the high street. Consider, for example, that food trucks and carts selling consumer packaged goods are apt to shift location from day-to-day (or even moment-to-moment) along a high street. In some situations, for example when selling services or subscriptions, sales associates may mingle directly with pedestrian traffic. For applications to these instances, the non-stationary retailer could be modeled as an agent in and of itself, with a dynamic and synergetic (service) journey path of its own that might interplay with (e.g., intercept and interrupt) customer journeys. To our knowledge, agent-based simulations of non-stationary retailers have not been published in the literature, although the problem of positioning non-stationary retail vehicles relative to urban points of interest (POIs) has been covered by location-allocation models (Murray 2018). It would seem, at least anecdotally, that agent-based models of urban parking behavior (Waraich and Axhausen 2012) might be useful in analyzing how non-stationary retailers such as foot trucks might consider locations, although in most cases these retailers require permits and the problem of locating space at the streetscape is relatively unique.

### Customer typology

There are natural affinities between typology-type approaches to customer journeys and agent-based modeling, where significant work already presents on classes and ontologies of agent characteristics and agent behaviors relative to urban processes and phenomena (Benenson and Torrens 2004), often at street-level (Torrens 2016a). Batty et al. (2003) discussed how the categorization of pedestrians must necessarily shift as the scale of observation of their behavior shifts. In other words, as you consider finer resolutions of space and time in human behavior, the commensurate typology of that behavior may need to be adjusted because people’s behavior takes on different foci at different scales of the city: “The law of large numbers also breaks down when the phenomena cannot be classified into categories from which general relationships can be inferred” (p. 674). Batty et al. (2003) therefore advocated for agent-based models that focus on mobility rather than location (p. 674). For our considerations of retailing, of course, *both* mobility and location are important, as shops are indeed tied to location, but they are also dependent on the mobility of high street customers at that location. Considered another way, Batty et al. (2003) essentially argue that at fine resolution one might bump consideration of behavior from the fixed structural characteristics of streets to the dynamic, mobile, and social tapestry of the crowds that populate them. This also implies that at fine-enough resolution, individual customers become relatively unique on a retail high street.

Significant progress has been made in customer journey modeling along parallel threads of inquiry into customer types. In some situations, the customer journey is considered as a high-level concept, used to differentiate the key agencies in customer journey systems, e.g., a service provider, the customer, and an intermediary that facilitates encounters between the two (Berendes et al. 2018) (p. 220). Underneath these classification hierarchies, customer journeys are usually considered for “typical customer behavior” (Berendes et al. 2018) (p. 219) and typologies can be (necessarily) rather simple. For example, Chen et al. (2019) developed a two-class typology of shoppers—those engaged in business or leisure shopping—in their eye-tracking study of customer journeys through airport retail concourses. One might conjure a much more “delicate” (Lee et al. 2013) (p. 902) typology of agents engaged in the customer journey; i.e., delicacy in the detail attributed to customer typology is particularly necessary when marketing efforts are being considered, for which fine-granularity targeting is often cost effective. These detailed classifications of customer journey types and of customer behavior are well suited to agent-based modeling; indeed, agent classes can be devoted to separating-out key differences in specific types of contextual customer agency from a backdrop of typical behavior. In this case, a base class of typical agency could be developed, with more detailed state and rule descriptors to handle specific agency.

For example, Lee et al. (2013) examined “malling behavior” in (indoor) shopping malls, i.e., categories of customer behavior in mall areas between stores. In mall contexts, as in high streets, it is important to note that not all behavior is focused on shopping, although much behavior has potential to transition to shopping. For example, in their survey, Lee et al. (2013) resolved distinct customer journey profiles for eating, “playing” (e.g., gaming), reading, resting, “seeing” (browsing), and shopping (p. 902). They were also able to delineate what they termed to be “store category selection” (i.e., visiting stores of a particular retail category) as well as phases of intra-store movement. Critically, Lee et al. (2013) were able to build a hierarchy of *behaviors* from these typologies. Although their work was not applied to agent-based modeling, it is perhaps straightforward to see how the hierarchical classification of customer behavior could be used to form agent classes as well as to build transition probabilities between agent behaviors within the context of economic events and conditions. The caveat, in mentioning these examples, is that most malls are indoors and well-controlled environments.

Similarly, conceptually-typological research has been undertaken to study outdoor retailing. Berendes (2019) introduced two value-based classes of customer in their work to examine high street customer trajectories. Berendes (2019) described utilitarian shopping (with an emphasis on efficiency, i.e., applying minimal time on the customer journey path for maximum price savings), and hedonic shopping (prioritizing enjoyment). Both types of shopping were considered as building value along the journey through experiential gains, e.g., through “idea shopping” (building knowledge about trends), “adventure shopping” (shopping-related search as recreation and socialization), and “exploring shopping” (shopping to gather information by browsing) (p. 315). Feng et al. (2020) discussed typologically-adjacent classification schemes for outdoor pedestrian behavior (of which customer journeys could be considered an important component) and they introduced a high-level classification based on behavior. This included strategic behavior, which takes place ahead of a trip and may have long-lasting consequences on the resulting journey, e.g., by determining the destination and activity schedule (p.2). A corollary for the customer journey would be shopping purpose and selection of a high street to shop on. “Tactical behavior” equates to route choice (p. 4) and involves distinguishing between types of spaces to be traversed, objects that may attract and repulse movement, information regarding the route as provided by signs, and the movement patterns of ambient pedestrians. Again, this typology is easily matched to the customer journey. For example, customers may choose routes through a high street based on distinguishing factors that include types of space (public sidewalks with road crossing, pedestrianized areas, public–private spaces such as outdoor malls); objects such as outdoor map displays and street furniture; and rhythms and motifs of ambient shoppers who may be walking around with large numbers of branded bags from a particular store or beverage containers with store logos. The relationship between tactical behavior and street signs is of obvious significance for retail high street customer journeys, which invariably take pedestrians past a large number of advertising and promotional signs as well as displays of retail goods. Lastly, Feng et al. (2020) described “operational-level behavior,” which they considered as pedestrian behavior that comes into play over small bundles of space and time in which pedestrians may need to dynamically react to and interact with conditions that they encounter. Feng et al. (2020) clarified that this can involve interaction with objects that attract, distract, obstruct, and repulse (p. 4); interaction with pedestrians; group behavior with pedestrians that share a “salient social identity and act according to the social norms of that group” (p. 4); and collision avoidance with proactive behavior to avoid future collisions. Many aspects of Feng et al.’s (2020) classification of operational-level behavior have matches in our consideration of touchpoints along the customer journey, including attraction to shops and the goods that they may display at the high street interface, interactions with greeters and sales staff at the entrance of a store, and social effects relative to peer customers in the crowd or egressing into and exiting from stores.

While the aforementioned typologies are largely concept-driven, there have been major inroads in developing empirical classifications of shoppers and journey classes. Millonig and Gartner (2011) used observational tracking, supported by GPS (outdoor) and *Bluetooth* (indoor) movement *tracing*, to develop data-driven typologies based on pedestrian movement characteristics. Their results have some synergy with existing typologies from consumer research [particularly the hedonistic/utilitarian typology introduced by Babin et al. (1994)]. Millonig and Gartner (2011) presented a very well-sourced typology of urban shoppers: they identified “passionate shoppers” (who stop often and for comparatively long times, mostly at fashion shops) (p. 13); “convenient shoppers” (who illustrate a higher average speed and display no significant preference for shops) (p. 14); “discerning shoppers” (whom they characterized like convenient shoppers, but with the added note that they like to frequent specialty shops) (p. 14); and “swift shoppers” (who do not stop often and usually visit food shops and supermarkets) (p. 15). Millonig and Gartner (2011) also used quantitative methods to data-match shopper types to utilitarian and hedonistic movement (p. 16–18).

### Shop choice and selection

Considered hierarchically, from wide area to hyper-local geography, the selection of high streets and shopping districts is at the top of the customer journey pyramid. By far the most steady stream of agent research into retail location selection has been developed by Timmermans and colleagues and that work has traditionally been based on agent-based implementations of trip and path-planning models that are “micro-simulated” to agents from methods traditionally used in regional science (Arentze and Timmermans 2007), activity-based models (Arentze and Timmermans 2002; Zhu and Timmermans 2011), motion planning (Dijkstra et al. 2011), network models (Han et al. 2011; Ronald et al. 2012), and time geography (Arentze et al. 2010). Such micro-simulation has the advantage of establishing a natural affinity between coarse-level models of urban activity dynamics, and fine-level considerations of shopper journeys within those systems (Dijkstra et al. 2013). But, in essence, micro-simulation is a scaled-down derivative of the original high-level model and in that sense does not usually treat realistic behavioral agency at the micro-scale of shoppers.

Other scholars have investigated shop choice and selection at (or within) this “micro-scale.” For example, Yoshida (2020) introduced an agent-based model for what they termed to be “shop-around behavior.” This might perhaps be best considered as a micro-simulation counterpart to the stream of location-allocation research for route selection developed by Timmermans and colleagues. Yoshida (2020) clarified that their approach attempts to move beyond two common threads of existing route selection models: aggregate (coarse) treatment of space, and Markov-type selection heuristics (inertia-based probabilistic trees) to account for pedestrians that are engaging in choice among routes. These deviations from the usual stream of research have important implications for agent-based models. Attention to fine-scale detail would imply that customers could be exposed to a huge number of choice-points while on the customer journey, and a deviation from inertia would suggest that the transition probabilities for agent states relative to those choice-points would need to be assessed at each time-step in the model (from a discrete span of time t → t + 1), for each discrete state, for each discrete agent. This would yield an absolutely massive state-space for the model to resolve, even for a limited parameterization of agents on a customer journey. Yoshida (2020) argued that this is necessary in shopping models because consumer behavior is very diverse (p. 123). Recalling the law of requisite variety: for a given model to produce faithful dynamics relative to actual high streets, there should be a match between model detail and the equivalent real-world behavior.

### Perception

As shoppers and would-be shoppers engage in a customer journey, their path through retail high streets connects them perceptually to their surroundings. Of course, many shops may have selected a high street location because people’s perception of that setting predisposes them to engage in consumption, e.g., themed food districts such as Brick Lane, or fashion districts such as Savile Row (both in London). For example, Lee et al. (2013) discussed how, in their study of indoor mall behavior, “customers spend much more time for visiting than moving” (p. 908). Agent-based models should be well able to accommodate these perceptual factors in simulation, because of their abilities to support sense–reason–act type exchanges between their state input and transition rule functions (Torrens 2018a). Roozmand et al. (2011) introduced a dedicated “perception module” (p. 1082 and p. 1084) in their agent-based model of customer decision-making. It was designed to accept spatial information and to assign meaning to that information, although the mechanisms by which this was achieved were not provided in the paper. In their *Format-Store* model, Mathieu et al. (2011) allowed agents to perceive signs in stores (through an interaction distance function), which they then used as targets of their movement. In this way, agent-customers “discover” inputs to their journeys through the store, with the result that “customers will therefore always take different paths, even when their shopping lists are similar” (Mathieu et al. 2011) (p. 125). Turner and Penn (2002) focused solely on the relationship between vision and the spatial configuration of built environment in their agent-based model based on space syntax (where “syntax” is interpreted as the logical structural progression of built space). Their approach was based on James Gibson’s idea for “natural vision,” i.e., that perception can be explained through the relationship between the environment and affordances, which are characteristics of the physical environment that facilitate physical behavior (Cutting 1993; Gibson 1950, 1966, 1979). Turner and Penn (2002) appealed for the research community to consider perception as a direct mechanism by which one might “regard the environment as the provider of possibilities rather than as a place to be rationalized” (p. 473). This plea actually speaks directly to issues that we consider in this paper regarding the customer journey. In other words, the retail high street manifests as a service-scape for customers that journey through it, with myriad retail-centered products and opportunities that are embedded within the happenstance of the adjacent social, historical, environmental, and technological substrate of urban streetscapes. Collectively, these factors combine to produce a perceptually deep setting for movement and custom. However, Turner and Penn (2002) were careful to conclude that their “results, though good, also show that a direct perceptual system does not suffice on its own” (p. 487).

### Cognition

While the features of retail high streets might help to (and in some cases be designed to) evoke perceptual contacts between pedestrians and service-scapes, the content and meaning of those touchpoints is often critical in helping retailers to align would-be customers’ cognition of their service offerings with the branding of those offerings. Once again, agent-based models have long been used to build cognitive models for pedestrian dynamics in urban context (Frank et al. 2001; Funge et al. 1999; Mohsenin and Sevtsuk 2013; Paris and Donikian 2009; Penn 2003; Raubal 2001b; Raubal and Worboys 1999; Stern and Portugali 1999; Torrens 2016b, 2018b), and it stands to reason that they could be useful for retailing.

Brown (1994) discussed how shopping has been related to work on cognitive maps: “Despite the inevitable variations from study to study, most analyses agree that retailing facilities figure prominently in mental representations of the city centre” (p. 553). For example, Brown (1994) (p. 553) referenced the famous work of Lynch (1960) theorizing that how people conjure their images of city environments might explain their movement, and research by Sieverts (1967) to discern first-order and second-order “cognitive shopping streets” in Berlin. These mental constructs could form the basis for driving the perception of agents in simulation (see our work (Torrens 2015, 2016b, 2018b) on driving agent motion by mental maps, for example). Brown (1994) also pointed out that large retail stores often feature prominently on people’s cognitive maps of cities (what Couclelis et al. (1987) referred to as “anchor points”). Brown (1994) was also careful to point out that customers’ cognition is intricately bound to their “attitudes towards and emotional involvements” (p. 553). In other words, while the customer journey may bring people into contact with retail touchpoints on the high street, it is customers’ own internal agency that determines the cognition that forms around that context. In his review of work by Van Der Hagen et al. (1991), Brown (1994) (p. 556) made a salient comment: that “the decision heuristics employed by shoppers are strongly influenced by the complexity of the extant retailing environment and the idiosyncrasies of the individual location.”

A pedestrian’s shopping motivation and goal can be construed as inducing and driving their cognition along the customer journey (Berendes 2019) (p. 315). Berendes (2019), for example, discussed that utilitarian customers may have pre-settled products that they are seeking out during the customer journey, while hedonically motivated shoppers may be comparatively more open to persuasion through marketing and other recommendation methods (p. 322). Aspects of motivation thus factor into how customers’ cognition of the retail high street shapes their customer journey. Consider, for example, that a utilitarian-motivated customer may frame the geography of the high street through a lens that prioritizes efficiency in movement and eschews distraction. A hedonically motivated journey, on the other hand, may factor-in the atmospherics of a streetscape, media from storefront displays, and the hustle and bustle of a weekend crowd as part of the experience that they are trying to garner from a high street journey. In this way, then, shoppers’ cognition of the streetscape is intricately bound to their shopping behavior, with marked expression in the way that they pursue the customer journey. For example, Yoshida (2020) introduced a useful distinction in shopping behavior that touches on issues of cognition. In Yoshida’s (2020) scheme, “planned action” was used to categorize shoppers’ behavior before they approach the high street: “itemizing of stores to be visited, working out of time budget, ordering of visit so that the visit is ‘somewhat efficient,’ which then informs the route to be taken.” (p. 125). By contrast, “unplanned action” is improvised: “a visit that occurs when something motivates a pedestrian to visit a facility without having chosen an errand in advance” (p. 125). Both may influence customer journeys: planned action may have a strong influence on the brand journey that is pursued, while unplanned action may determine how much of the retail high street shapes that journey once enacted.

While it seems straightforward, at least conceptually, that connections between cognition and the customer journey could be accommodated within agent-based modeling frameworks, we did not find much existing work on the topic. Roozmand et al. (2011) introduced an agent-based model that treated customer decision-making as a function of culture, personality, and “power distance.” In doing so, they expanded on Costa Jr. and McCrae’s (1992) factorial model of personality, social status, and social responsibility (which actually was designed to examine personality disorder) (Roozmand et al. 2011) (p. 1075). They also built on Wilber’s four-class model of consciousness: interior-individual characteristics (desire, drive, emotion), exterior-individual conditions (body and objects), interior-collective states (common knowledge and norms), and exterior-collective factors (the environment and social structures) (p. 1080). Of interest to our paper is that the approach used by Roozmand et al. (2011) mapped these socio-behavioral factors to state update schemes in their agent-based model as part of the activation component for agent decision-making (p. 1075).

It is also salient to mention that retailers themselves also use (their own) cognition of the high street in determining their service-scapes. In a study of retailers’ mental maps of shopping areas, Brown (1987) detailed how the managers of stores view the local retail environment. Brown (1987) discovered that retailers were most aware of magnet stores, then the complementary or non-complimentary associations with adjacent stores, followed by proximity to traditional sources of customer traffic such as parking facilities and offices. Brown (1987) also settled on a 200 m “maximum perceived distance” within which retailers considered generating compatible footfall custom.

### Path-planning

Path-planning involves the selection of a course of movement, usually between an origin and a destination or satisfying a chain of destinations. As we discussed earlier in the paper, the physical paths that customers and would-be customers traverse through high streets is one of the central concepts in considering retail customer journeys. Knowledge regarding how customers settle upon choosing particular high street paths is therefore incredibly useful for a variety of retail operations, from consideration of where to site a store to deciding where to place visual advertising. Path-planning is reasonably well developed in agent-based modeling, following decades of work on the problem of robot motion planning (Latombe 1991). It is often framed as a problem of modeling accessible paths through urban settings, and the likelihood of individuals to select among an assortment of paths can be decomposed to very efficient heuristics from computer science, which often take minimal parameterization to evaluate (Dijkstra 1959; Hart et al. 1968). Key in most agent-based path-planning models is that one may assume that a pedestrian is motivated to select a shortest path. However, this is not necessarily the case on retail high streets, where other factors beyond minimizing walking distance understandably come to the fore (Bitgood and Dukes 2006; Garbrecht 1971). Millonig and Gartner (2011) articulated this point well in their examination of wayfinding behavior among shoppers: “For pedestrians, the shortest path does not always represent the optimal route for an individual’s purposes, as studies have revealed that people often forgo to take the shortest path and prefer the ‘most beautiful’, ‘most convenient’ or ‘safest’ path.” (p. 3). Moreover, pedestrians have many degrees of freedom in their movement through urban streetscapes and their paths may often become highly dynamic, reactive, and adaptive, bucking the simple drivers that computational heuristics may suggest as proxies for their planning behavior (Torrens 2016a).

Nevertheless, path-planning agents can be useful in estimating coarse flows of pedestrians between fixed points on a retail high street (for example, between transit stations and anchor stores). Data to parameterize path-planning are often readily available: the number of pedestrian trips that originate at a parking garage, for example, may be available from ticketing kiosk data and the number of people that enter the front door of a store is usually known to retailers. Estimates of the likely flow of pedestrians between these locations can be built from input–output models or from spatial interaction models, leaving potential paths that they may have traversed open to estimation by path-planning heuristics, e.g., greedy search on graphs that represent streets as edges and stores and high street points of interest as vertices.

Turner and Penn (2002) presented a compelling criticism of traditional heuristics for path-planning in agent-based models (which usually rely on graph-based traversal cost functions to drive shortest-path-planning), which is relevant to consideration of specifically *customer*-centered paths. They posed the question as to whether it is “really plausible that the human brain continually reassesses an internal cost function, or is it that the human in led by less tangible factors—her curiosity or his desire?” (p. 474). They also went on to argue that in overlapping cost environments (such as high streets, which have temporal costs to move through, costs in effort, costs in distraction, costs in exposure to noise, costs in nuisance of crowds, etc.), it is difficult to separate costs. [Recent research in transportation planning has actually begun to explore how the multilayer nature of path heuristics might be accommodated within a cost structure: see the regret-minimization approach by Chorus et al. (2008) and the level-of-effort approach built into agent-based pedestrian walking models by Guy et al. (2010).] Turner and Penn (2002) argued that human movement is “natural movement” (p. 474), driven by affordances rather than the object-based approaches popularized in agent-based modeling that mimicked heuristics from robot motion planning (Latombe 1991, 1999).

### Foot traffic

The volume of foot traffic along a retail high street or along sections of a street is usually estimated using some form of spatial interaction model (Daamen and Hoogendoorn 2004), which explains aggregate flow in terms of mass parameters that describe a source generating a flow (e.g., a transit stop or a parking garage) and a sink that attracts that flow (e.g., a particular retail store location), the physical or network distance between the source and sink, and some expression of the friction that can act on that distance to dissuade customers from making journeys of an excessive length. There are numerous theoretical and observational supports for estimating foot traffic flow in these terms. Brown (1994) described the work of Morris and Zisman (1962) to empirically measure pedestrian movement in Washington D.C., which revealed the strong relative influence of traffic to retail from nearby offices (p. 555). Brown (1994) also highlighted a similar conclusion from Ness et al.’s (1969) examination of lunch-time pedestrian traffic in Toronto (p. 555). Nelson (1958) introduced the “rule of retail compatibility” and “theory of cumulative attraction” (see Brown (1994) (p. 554–555) for a discussion). Respectively, the two notions conceptualize the common observation that compatible retail stores can often generate cross-traffic of customers between them (compatibility) and that stores can take advantage of shoppers’ habits of comparing similar goods [termed as “matching” by Brown (1994) (p. 562)] by siting stores of similar retail trade categories in proximity (cumulative attraction). In these two cases, stores essentially generate their own foot traffic dynamics (in terms of aggregate flows) within the micro-scale of the retail high street, and the specificities of the actual shopping experiences and service-scape (above and beyond their trade classification relationship) will explain the fine details of what form any flow among them may assume.

### Wayfinding

Waypoints, considered generally, are points in space and time that people consider when planning and executing their navigation. Wayfinding (moving between waypoints, usually by navigating) is perhaps essential to understanding the customer journey (Dogu and Erkip 2000; Gärling and Gärling 1988), as visits to individual stores along the high street shopping trip constitute the significant touchpoints along the entire journey chain. Other significant waypoints along the customer journey could be identified between visits, including street signs, advertising, information kiosks, and features of the high street urban design such as pedestrianized areas, plazas, malls, and so on (Hess et al. 1999; Moudon et al. 1997). Wayfinding has been well treated in geographical applications of agent-based modeling (Frank et al. 2001; Hajibabai et al. 2007; Raubal 2001a, b, 2008; Torrens 2016b, 2018b). There would seem to be some straightforward connections between the work of geographers in this area and interests in customer journeys and touchpoints. For example, consider that waypoints constitute both touchpoints in people’s behavioral geography and in the organization of their movement (wherein the waypoint essentially allows them to check-in with the territory they are moving through) and with the service-scape (e.g., when high street’s offer “you are here” maps (Meilinger and Knauff 2008) of retail sites, often presented by trade type). Although not directly focused on modeling and simulation, Millonig and Gartner (2011) presented a very detailed review of the sorts of location-based data that are available for the support of wayfinding tools for shopping decision-support systems.

### Steering

Steering is a large focus of many commercial customer journey mapping systems that work indoors. Generally, such systems use closed circuit television (CCTV) cameras to identify and track individual customers as they move around within a store. The end result is either a “heatmap” or a map of movement traces. These traces essentially illustrate how customers traverse the store by steering as a response to path-planning, locomotion, and interactions with staff and products. The topic of steering is very well covered by agent-based modeling. With the exception of Alasdair Turner’s (Turner et al. 2001; Turner and Penn 2002) work on examining steering in indoor art galleries, our review did not return academic work on steering-based agency for retail environments. It is worth mentioning that many pedestrian-based agent models rely on Helbing’s social force model (Helbing and Molnár 1995) to produce steering in crowded corridor-type settings such as sidewalks. Turner and Penn (2002) are critical of “social force” type approaches to collision avoidance, as is Torrens (2012; Torrens et al. 2012). Turner and Penn (2002) put their argument elegantly when they ask, “does the corporeal human bump through a crowd of corporeal humans, or does the human guide him or herself through gaps in the crowd?” (p. 474).

### Proximity

The relevance of touchpoints in docking the customer journey to retail service-scapes brings to the fore the notion that proximity effects may be critical factors in how touchpoints interplay with pedestrians on retail high streets. For example, pedestrians are generally understood to maintain distance from physical objects to avoid collisions (Basili et al. 2013; Collett and Marsh 1974; Cutting et al. 1995; Huber et al. 2014; Kitazawa and Fujiyama 2010), and a personal distance from other people that variously relates to personal and social factors (Adams 1995; Aiello and Thompson 1980; Altman 1975; Hayduk 1983). When encountering touchpoints along a customer journey, pedestrians on a retail high street may depart from their adherence to these buffering effects: they may seek-out contact with things in the retail service-scape. Indeed, the locations along a high street in which pedestrians deviate from norms of collision avoidance—to patterns of contact-seeking along the high street—may be incredibly valuable information for retailers, indicating, for example, where visual advertising might resonate with pedestrians as they pass by [e.g., the spatial reach and temporal reach of outdoor advertising media (Bhargava and Donthu 1999)]. However, separating-out the contextual situation for proximity-based buffering in crowded and often hectic high street scenarios may be difficult. Our review of the literature did not return examples of agent-based modeling being used to examine links between proximity effects and customer journeys. Nevertheless, proximity effects between moving pedestrians in crowds and on street scenes have been well covered in agent-based modeling for applications in animation and special effects and these types of applications could feasibly be adapted for examining journeys on retail high streets. Proximity-driven movement is often used in motion control of animated characters in computer graphics and especially in computer games, where geometry can be employed to produce realistic-looking crowd patterns for large numbers of agents atop very efficient data structures (Stüvel et al. 2017). Adaptive roadmaps (a modification of probabilistic roadmaps used in robot motion planning) are computationally efficient structures for building personal space buffers between agents while they pursue navigation graphs in heavily animated graphics scenes (Gayle et al. 2009; Sud et al. 2007). Proximity effects are usually achieved using some variation of spatial tessellation between agents, based on their relative positions in a scene, while also taking into account the likely movement path that they will take through the same scene (Kavraki et al. 1996). The tessellation can straightforwardly be associated to agency, to account for example for an agent’s attraction to an object or its adherence to personal buffering space in a given street context (Torrens 2016b).

### Locomotion

Locomotion is a critical component of the customer journey along retail high streets. That people are in motion along a high street customer journey can be useful information for retailers, as can determination of where, when, and with whom that motion is taking place. Similarly, certain types of locomotion (how people articulate their motion through stride, body language, how they walk while holding shopping bags, etc.) may suggest shopping behavior and actions, as when customers are walking past a store front and slow and begin to gaze at the displays (Burgoon et al. 1986; Neider et al. 2010; Patla 2004; Shimojo et al. 2003). Customers may also display different locomotion patterns that could indicate their demographics (consider, for example, how locomotion differs between children and the very elderly). Our review could not account for much published work that would explore the relationship between high street customer journeys and locomotion. Nevertheless, the topic presents an interesting opportunity for agent development. Locomotion is very well covered in agent-based modeling in animation and computer graphics (see Pelechano et al. (2008) for a detailed review of the field). It is less frequently used in computational social science or even in geography, where attention is usually placed on movement (we may distinguish movement such as path-following, navigation, wayfinding, steering, collision detection and avoidance from motion such as stopping, idling, leaning, reaching, and so on) (see Torrens (2016a) for a review).

We might consider the absence of locomotion and its relevance to the customer journey. Many aspects of the retail journey involve non-movement. Indeed, the parts of the service-scape that allow retailers to take advantage of customer’s behaviors to stop, pause, rest, queue, idle, etc., often align with touchpoint opportunities. Consider, for example, when a customer is walking purposefully along a retail high street but suddenly comes to a stop outside a store display. This example is a canonical opportunity for a retailer to evaluate the effectiveness of that display touchpoint: if a customer subsequently enters the store, the retailer may assume a connection between the display and a subsequent purchase registered at the point of sale. A simple query, of the like, “what brought you into the store today?” may provide enough information to formalize that connection. Lee et al. (2013), in studying (indoor) mall behavior, presented a method for detecting periods of “stay” in customer journeys by analyzing sessions of relative inaction via Wi-Fi localization (so-called Wi-Fi fingerprinting). In essence, their approach used movement traces from Wi-Fi to isolate rough areas of non-movement for customers in indoor shopping malls. Their test cases demonstrated that customers spend more time in browsing and contemplation type activities than they did in moving.

### Group dynamics

High street retailers are often interested in groupings of people as they traverse the customer journey. Some of these groupings may be straightforward, as in the example of tour groups that might be led on high street walking tours by a tour guide. In other instances, retailers may be interested in isolating the customer journeys of particular groups of people from the general motif of foot traffic along the high street, e.g., groups of young people walking together past fashion stores. Batty et al. (2003) raised the issue of treating group-level phenomena on streets; they noted that “There is also the somewhat mystical property of large crowds being formed with their own momentum which binds them together and drives their movement. … such herding instincts due to identity of purpose—‘crowd fever’ so-to-speak” (p. 675). They went on to comment, however, that, “There is little descriptive material on which good models of these dynamics might be built, and the interpretations that do exist are not found within mainstream geographical, urban, or architectural analysis.” (p. 676). Regarding the high street customer journey, we might build understanding of group dynamics in two ways that associate directly to retail service-scapes. First, we may consider how customer journeys group at or in the vicinity of touchpoints. Batty et al. (2003) actually mentioned a related phenomenon when discussing crowding events at high street carnivals in London: “Canetti (1962) describes such events as being highly focused on single points of attraction which are spatially associated with agglomerations of individuals.” (p. 676) (Canetti 1962). This notion is already covered in agent-based modeling of pedestrian movement in the outdoor environment, usually based around physics-inspired models that can focus movement patterns around well-understood models of attraction and related continuum mechanics (Treuille et al. 2006) of particle-based crowd motion (Helbing et al. 2000; Liu et al. 2014; Schweitzer 1997). Second, one might perform a *grouping* of customer journeys based on particular attributes (of the people making those journeys, or of the journey geography that they express). This thread of research has also been picked-up in agent-based modeling of pedestrian movement (Nara and Torrens 2007; Torrens et al. 2012), using in particular trajectory-based classifiers to build typologies of movement paths for synthetic agents, with some experimentation to match those classifiers to real-world urban scenes (Nara and Torrens 2011).

### Sociality

The social phenomena that people generate are an essential component of retail high streets. As Bartelheimer et al (2018) remarked: “customer experience accrues over time and is also co-created with other actors” (p. 3). Bartelheimer et al. (2018) made a passing (but very relevant) comment in his paper on community platforms for retail: that “Service ecosystems emerge and continuously adjust themselves based on shared institutional logics, such as actors sharing a common belief system regarding high streets” (p. 3). If we also consider that at least an ordering of retail shopping streets is a recurring feature of people’s cognitive maps of city environments, we begin to see conceptually that we might be able to establish a set of shared cognitive attributes for high streets generally, or even trends regarding specific high streets that are exchanged through the social actions of shoppers on their customer journeys. This is useful for agent-based modeling, in particular, which can use those attributes to form rules. Indeed, agent-based modeling of belief systems is well covered in much of the computational social science literature (Epstein 2007) (see work on belief–desire–intent (BDI) models to drive synthetic pedestrians in simulation (Ronald et al. 2007), for example). As a caveat, we mention the observation by Siebers et al. (2014) that, “in ABM, although most models have been inspired by observations of real social systems they have not been tested rigorously using empirical data and most efforts do not go beyond ‘proof of concept’” (p. 101). As Bartelheimer et al. (2018) pointed out, “there are little (if any) attempts made in research to establish or study social shopping communities in local high street retail” (p. 5). Our review for this paper returned very little research on these social aspects of retailing outdoors; there was also a dearth of existing research on how one might model social aspects of customer journeys indoors.

### Fraud and customer journeys

Loss prevention is a major concern for retail operations. Ustun et al. (2006) provided some figures for the cost of fraud to retailers: in 2004, US retailers lost an estimated US $9.1 billion to shoplifting (p. 365), and in the UK, retailers invest US $750 million in security systems annually. As Ustun et al. (2006) described, security is costly for retailers, and understanding the journey that shoplifters might take through a store can assist in estimating likely returns for different security measures along that journey path. This is of relevance to our discussion of high street retailing straightforwardly: if somebody walks out of a store without paying for a product in their possession, it may be grounds for accusing them of theft. Some work on agent-based modeling of shoplifting has been published, and aspects of the research that it represents are relevant to customer journeys. Ustun et al. (2006) introduced a conceptual model, based on an agent methodology, for simulating physical security in shops (supermarkets). Although their model is based on indoor security, it maps neatly to customer journeys, taking into account the physical layout of the store, as well as the (visual) reach of fraud-detecting sensors through the same environment, as well as the paths that security staff routinely take through the store. Lopez-Rojas et al. (2015) introduced an agent-based model, *RetSim*, that was sourced in sales data to identify fraud in interactions between sales staff and customers of a Swedish shoe retailer (fraudulent use of coupons, returns, and voided sales). An interesting proposition would involve examining customer journeys on outdoor high streets for loss prevention purposes, e.g., customer journeys of shoplifters that pass through multiple stores. We have not seen work of that nature in the published record, although at least anecdotally we are aware that security staff on high streets do coordinate to identify and share information about shoplifters.

### Time

The ability to model time (and related dynamics) is a central component benefit of agent-based modeling (Torrens 2009). Time is obviously crucial in considering the customer journey and in mapping journeys to shops’ service-scapes. For example, last-meter logistics of loading and unloading on the high street must often be managed relative to the timing of opening hours and customer rhythms so as not to interrupt the customer experience by physically disturbing customers’ journeys (Glaser 2016). Also, high street retailers are often sensitive to collective timing of customer journeys, particularly surges in visits. Retailers therefore have an interest in tracking the rush hour dynamics of commuting along the high street, trips to and from school, as well as lunch hour dynamics for nearby offices (Hess et al. 1999; Moudon et al. 1997; Ness et al. 1969). In many instances, proximity to other retail sites and their hour-by-hour or weekly dynamics are important for determining likely customer journeys to other stores: examples include traffic to banks and ATMs on Fridays (pay-day) and theater and movie opening times for restaurants. *Within* the customer journey, timing can also be useful, for example in singling-out the spaces and durations of dwell time, as well as aspects of efficiency in how quickly customers move in and out of a store from one entrance/exit to a high street or another (Spearpoint and Hopkin 2020). For retailers that work with relatively narrow profit margins (such as high street convenience stores selling consumer packaged goods), frequency of transaction is critical to their relationship to customer journeys and so even small savings in transaction time can be actionable (Antczak and Weron 2019). Similarly, advertisers may be interested in determining which parts of the high street customer journey are taking up lots of time; slow-moving crowds of customers may yield a lot of views past physical displays, for example (Garaus and Wagner 2019). While time is well treated in almost all agent-based models, our review did not uncover much work that specifically focused on time as a simulation scenario for consideration of outdoor retailing. Antczak et al. (2020) recently introduced a *NetLogo*-based (Wilensky and Rand 2015) agent model of queuing within supermarkets, tied to point-of-sale data.

### Atmospherics

Atmospherics are often a significant part of the customer experience affecting indoor journeys. These most commonly relate to lighting (Custers et al. 2010), aroma (Morrin and Tepper 2021), product placement (Tan et al. 2018), and design features (Stevens et al. 2019), among other factors. Atmospherics are also important in outdoor high streets; indeed, the atmospheric character of a high street setting may contribute to retailers’ decisions to site their facilities there (Goffmann 1963, 1971). Aspects of high street atmospherics could be directly adapted from indoor store considerations. For example, Choi et al. (2006) considered connections between street lighting and space syntax, and Omer et al. (2015) examined connections between different types of street layout and agent-based movement in simulation. Satoh (2021) has recently introduced an innovative scheme for pairing customer journey to smart signs in a way that allows for the effecting of atmospherics within a store. Satoh (2021) described a digital sign that used radio frequency identification (RFID) sensors to build a contextual and spatially-bound record of a customer’s interaction with it (as a touchpoint) (p. 71). The system then uses an agent running on the local system to activate programs to drive signs in the vicinity. It is straightforward to see how Satoh's work could be extended to the high street and adapted for street-facing signs, for example. Interestingly, the agent programs “migrate” (p. 72) between the touchpoint artifacts through communications, without having to negotiate centralized servers within the broader the retail information system (p. 73). The system is implemented on reasonably light technology: Satoh (2021) demonstrates the concept on *Raspberry Pi* devices (p. 78). Akhter et al. (2019) introduced a scheme for counting pedestrians on streets using infrared sensors embedded in smart city type systems, which are designed to detect pedestrian humans from a backcloth of dynamic street activity.

### Artifacts

In many high street retail settings, the service-scape is landmarked with artifacts. These include outdoor seating areas for dining, street-facing counters, vending machines, outdoor displays and racks for goods, and information kiosks such as “you are here” maps. In these cases, artifacts extend the retail service-scape into the public space of the sidewalk, drawing the retailers’ operations into direct contact with customer journeys. In this way, then, we might consider some artifacts as key touchpoints in the customer journey. For example, through questionnaire surveys, Lee et al. (2009) examined customer use of self-service kiosks and information kiosks (indoors). They concluded that general trends in customer patronage at retail kiosks were related to the experience that was enjoyed at those artifacts. How customers interact with high street artifacts, and then go on (or not) to visit stores would seem to be an area that would be straightforwardly explored by agent-based modeling. We were not aware of any such work in our review. However, Batty et al. (2003) discussed the significance of objects on the streetscape in building their agent-based model of festival goers. They found that at a small-scale (a high resolution of the high street), “elements or objects vary in such a way that temporal dynamics are intrinsic to their representation and explanation” (p. 673).

### Location-based services

Many retailers are now experimenting with service-scapes that manifest in the omnichannel that forms at the hybrid of material and virtual retailing. It therefore makes sense that retailers would consider the customer journey as being co-determined between the two. Sometimes this connection straightforwardly separates out pre-purchase components of the customer journey and post-purchase stages. For example, many high street stores facilitate the purchase of goods online with in-store pickup. In the same way, Online stores may accommodate the purchase of goods through their virtual platforms, with options to physically collect them at another retailer’s tangible presence on the high street. More sophisticated connections are also possible across the omnichannel. For example, location-based services that function on top of location-aware technologies that commonly feature in the devices that we carry while moving on the high street facilitate a range of customer touchpoints that may flit between the physical components of customer journeys and their digital counterparts, as well as novel techniques to use digital technologies to augment physical journeys (Niantic Labs 2016).

Millonig and Gartner (2011) have explored the connection between the positional data that location-aware technologies yield and the movement and inferred interest locations (which we could consider as touchpoints) of outdoor shoppers. Their fieldwork to examine the location data of shoppers using spatial clustering analyses showed that inferred movement could be used to classify shopper types (“passionate,” “convenient,” “discerning,” and “swift”) (p. 13–15). In some ways, these types also confer customer journey attributes: customers engaged in discerning shopping may be more apt to browse window displays along a high street, for example. Further, coupled with interview data, Millonig and Gartner (2011) used spatial analysis to build estimates of shopping trip types (“utilitarian” and “hedonistic”) (p. 16–18).

Another stream of agent-based modeling is focused on the development of location-based services *as shopping agents*. This represents an approach that focuses on the design of broker-type agents [“agent intermediaries” (Tewari et al. 2003)] that serve as shopping assistants on location-based commerce platforms. They are of high relevance to our discussion of customer journeys, because the broker agents are usually tasked to poll high street stores’ inventory systems [what is often termed as “location filtering” (Fano 1998)] to present product availability and pricing to customers via their smart devices, but while they are physically engaged in a customer journey. Various location-based services have also been considered for delivering advertising and coupons to smart devices and customers as they move. Research-oriented systems include *Easishop* (Keegan et al. 2008), *Impulse* (Tewari et al. 2003), and *Shopper’s Eye* (Fano 1998).

### Staff-side agency

Staff are a critical component of the service-scape in most indoor customer journeys. In many cases, staff will physically introduce themselves into customer’s paths when they recognize key aspects of a customer’s journey behavior. For example, staff may identify that a customer is searching for goods and offer to assist in identifying the product location. Staff may notice that a customer has a hurried path and offer to assist them in checking out quickly. Shop staff may recognize that a customer journey has taken that customer back to a display multiple times and determine that they have an opportunity to influence a sale. Further, security staff may identify unusual customer journey paths of shoplifters. Agent-based models have been used to explore staff interactions with customer journeys. Of particular note are a series of immersive game environments that allow staff to train with virtual customers (with each customer programmed as a non-player character (NPC) agent). Mathieu et al. (2011), for example, introduced the “*Format-Store*” environment to assist training shop staff for a variety of interactions with customer journeys. Again, we should mention that this model is designed for in-store training, rather than high streets, but we note it here because of the innovation that it introduces, and because it raises several open research questions about how staff-side interactions might interplay with customer journeys outside the store to extend the service-scape from the inside to the outside. *Format-Store* was developed as a three-dimensional (first-person gaming type) environment, focused on providing virtual customer scenarios (mostly effected through chat-bot type textual interaction) for customer relationship management (solving customer problems, providing necessary information to customers when requested), managing the store’s physical environment (spill hazards and store lights flickering), and restocking products when shop floor inventory runs low (p. 120). Mathieu et al. (2011) discussed how the gaming environment can assist in experiential learning, particularly in providing what they term as “in situ” learning. Key, in providing realistic situational scenarios, is the development of what we would recognize as customer journeys for the NPC agent-customers in *Format-Store*: “The customers wandering in the store at any time are merely going to their business—shopping for goods—trying to fulfill internal goals—purchasing items on a shopping list or querying for information—instead of following a scripted behavior.” (p. 122). A particularly innovative aspect to their treatment of simulated customer journeys involved the introduction of “disturbances in the environment” (p. 122), e.g., parameterizing virtual customers with missing information, blocking parts of the store with spills, removing informational cues such as signs, etc. In essence, the model agents were designed with the capability of interrupting the (simulated) customer journey, thereby allowing for training on the staff-side of the service-scape to address how staff can respond. The virtual customers in the model were designed to exhibit traits of being upset and complaining, for example.

## Some near-term research directions for urban economists

In the preceding text, we have reviewed the concept of the customer journey as originally developed for in-store and indoor retail analysis of the customer experience. We have argued that the same concepts could be usefully deployed to outdoor components of the customer journey, extending the reach of retailers’ analysis capabilities to the urban environments and urban economies that sit outside their stores on the streetscape. We have also argued that many components of the customer journey framework lend themselves relatively easily to representation in agent-based models. This is meaningful, because routine dynamics of high street environments may contain many phenomena and conditions that are largely unknown or unknowable to retailers; agent-based models provide a promising opportunity for retailers to experiment with facets of customer journeys that might evolve, pre-transaction, before a customer reaches a store, or, post-transaction, after they reach the store.

It is also perhaps straightforward to believe that agent-based models could be used—holistically—for example, by business improvement districts, alliances of high street retailers, chambers of commerce, and urban planning agencies, to explore how high street customer journeys may interplay with their designs, plans, and policies for downtown environments. There are ample opportunities for cross-disciplinary research to examine the linkages between high street customer journeys and issues of land-use and transportation connections (for example, congestion pricing). Given the relatively rapid turnover of high street store occupancy in many cities, agent-based modeling could be usefully tied to examination of vacancy rates and spill-over and contagion effects in local community economies. For example, in New York City, retailing was recently responsible for 344,600 jobs across 32,600 businesses, generating $16 billion in wages for the city and $55 billion in taxable sales to New York City (Office of the New York State Comptroller 2020).

We might note the current relevance of this possibility as we write this paper in the middle of a global pandemic that has severely impacted retailing and downtown vitality in many cities, due to loss of foot traffic along streetscapes as customers sought to minimize face-to-face contact in crowded settings (Sayyida et al. 2021), interruptions to the labor supply (Ganong et al. 2020), and difficulties in maintaining retail supply chains (Aryapadi et al. 2020; Burgos and Ivanov 2021; Sharma et al. 2021). During the pandemic months of March to May, 2020, taxable sales for the retail sector dropped by a third (Office of the New York State Comptroller 2020). Bitterman and Hess (2021) have recently put forward the viewpoint that some retail high streets may “go dark” and essentially not recover from the pandemic. This view ties into a burgeoning debate about what cities and urban economies may look like in the post-pandemic city. Bitterman and Hess's (2021) use of the term “go dark” is clever, as it suggests both that the lights along high streets may go off as stores close operations, but also that new forms of retail operations may emerge in their place, as “dark stores” (Morgan 2020). Dark stores are former high street retail premises that have been temporarily nudged into new uses to build revenue during the pandemic, traditionally as hyper-local distribution sites. This is tied to the growing emergence of app-based delivery platforms for retailing. Dark stores also have broader implications for urban design and planning, through concepts such as the 15-min city, which proposes the idea that cities could form around activities that fall within small space–time prisms of access to housing, employment, retail, and urban services and amenities. Moreno et al. (2021), for example, have tied the 15-min city directly to consideration of what cities could look like post-pandemic. Other futures for the retail high street are more upbeat. In New York City, for example, the “Open Store Fronts” initiative was launched as a response to the pandemic’s impact on brick-and-mortar retailing. The scheme opened-up previously public areas of the streetscape (sidewalks, bike lanes, parking spaces, and some road segments) directly to retailers’ use (New York City Departmet of Transportation 2021). This initiative has directly extended retailers’ service-scapes to the high street and essentially forces into consideration much of the hypothetical propositions that we have examined in this paper.

## Conclusions and outlook

In this paper, we have reviewed the potential mappings between agent-based modeling and the customer journey along retail high streets. In our review, we discussed existing support for several aspects of the customer journey is agent-based modeling frameworks. We also identified other factors of the customer journey that might be supported by agent-based modeling (along twenty discrete axes of agency), and this indicates several promising avenues for future research inquiry.

Much of our review has highlighted outdoor characteristics and considerations of the customer journey, while our review of agent-based modeling for retail applications has largely focused on indoor customer journeys, leading to a discussion of how indoor models might be alternatively considered for outdoor applications. Inevitably, we have turned to models of general pedestrian processes and phenomena to tease out possible connections between high street customer journeys and retail service-scapes. This is quite simply due to a relative lack of modeling work for the outdoor settings of retail, relative to indoor environments. As we have already mentioned, retailers generally have much more available information about the indoor context for the customer journey, because it takes place within their stores, where retailers have considerable access to data and observational methods. Outside, on the high street, retailers must cede much of their awareness of the customer journey to the background motifs of day-to-day streetscape dynamics. Nevertheless, the outdoor customer journey is critical to retail success, and specifically crucial in building custom for high street retailers. Our review highlights several promising points of connection between existing understanding of the indoor customer journey, outdoor factors of high street journeys, and the broad swath of existing research work that has been performed to build agent-based models of pedestrians in dense urban streetscape settings. Agent-based models are particularly well suited to representing the customer journey on high streets, where retailers must almost necessarily rely on what-if type queries and suppositions to examine how they might actionably map their outdoor service-scapes to customer journeys.

Clearly a recurring limiting factor in developing closer synergy between agent-based modeling and high street customer journeys is data. The practicalities of capturing actionable information from real customer journeys, encapsulating it into an agent-based framework, populating a dynamic simulation that can sufficiently contextualize dynamic retail high streets and the retail operations that rely upon them, and then collating useful insight for retailers are, as one can imagine, more nuanced than conceptual models suggest. The difficulty of observing and of building insight about moving and interacting pedestrian customers is a central limitation to advancing work in this area. Some of the data limitations discussed by Feng et al (2020) in relation to pedestrian modeling are relevant, particularly that large and complicated pedestrian scenarios (of which we could certainly consider retail high streets as a canonical example) are relatively under-represented by existing data products, that there is scant empirical evidence for simultaneous and multi-dimensional treatment of pedestrian behavior, and that collected data are not usually generalizable to other scenarios (p. 1). It would therefore seem appropriate that systematic data survey instruments might be established on retail high streets to address this. Very promising work in this area is advancing. The review and survey results presented by Millonig and Gartner (2011) provide a thorough overview of what is possible in public spaces through location-aware technologies. Significant data products are also available in private form directly from retailers, particularly as a by-product of the drift of e-commerce analytics into tangible retailing. It is for example no longer necessary for customer experience analysis to examine “typical customer behavior” [(Berendes et al. 2018) (p. 219)]; it might instead begin to study very specific customer behavior, where retail operations can be more effective. Ultimately, agent-based models would seem to be highly useful tools for generating these data. These could be entirely synthetic data, used to test conceptual ideas. They could also be empirically grounded simulation data, in which agent-based models could be parameterized around known data-points (perhaps even data from digital touchpoints in the high street) and animated relative to real service-scapes. Indeed, much of the e-commerce platforms that high street retailers now compete against are already running on agent-based artificial intelligence schemes (Fasli 2007).
